# Protocol for Biospecimen Collection and Analysis Within the BACPAC Research Program

**DOI:** 10.1093/pm/pnac197

**Published:** 2022-12-16

**Authors:** Aaron J Fields, Stefan Dudli, Andrew Schrepf, Angie Kim, Bernice Pham, Estefania Gallego, Sandra Mendoza, Sharon B Meropol, Jessa Darwin, Gwendolyn Sowa, Nam V Vo

**Affiliations:** Department of Orthopaedic Surgery, University of California, San Francisco, California, USA; Center of Experimental Rheumatology, University Hospital Zurich and Balgrist University Hospital, University of Zurich, Zurich, Switzerland; Department of Anesthesiology, University of Michigan, Ann Arbor, Michigan, USA; The New York University Langone Health, Center for Biospecimen Research and Development, Office of Science and Research, NYU Grossman School of Medicine, New York, New York, USA; The New York University Langone Health, Center for Biospecimen Research and Development, Office of Science and Research, NYU Grossman School of Medicine, New York, New York, USA; The New York University Langone Health, Center for Biospecimen Research and Development, Office of Science and Research, NYU Grossman School of Medicine, New York, New York, USA; The New York University Langone Health, Center for Biospecimen Research and Development, Office of Science and Research, NYU Grossman School of Medicine, New York, New York, USA; Department of Population Health, NYU Grossman School of Medicine, New York, New York, USA; Department of Physical Medicine and Rehabilitation, University of Pittsburgh School of Medicine, Pittsburgh, Pennsylvania, USA; Department of Physical Medicine and Rehabilitation, University of Pittsburgh School of Medicine, Pittsburgh, Pennsylvania, USA; Department of Orthopaedic Surgery, Ferguson Laboratory for Orthopaedic and Spine Research, University of Pittsburgh, Pittsburgh, Pennsylvania, USA; Department of Orthopaedic Surgery, Ferguson Laboratory for Orthopaedic and Spine Research, University of Pittsburgh, Pittsburgh, Pennsylvania, USA; Department of Orthopaedic Surgery, University of Pittsburgh School of Medicine, Pittsburgh, Pennsylvania, USA

**Keywords:** Chronic Low Back Pain, Biomarkers, Biospecimen, Omics

## Abstract

The Biospecimen Collection and Processing Working Group of the National Institutes of Health (NIH) HEAL Initiative BACPAC Research Program was charged with identifying molecular biomarkers of interest to chronic low back pain (cLBP). Having identified biomarkers of interest, the Working Group worked with the New York University Grossman School of Medicine, Center for Biospecimen Research and Development—funded by the Early Phase Pain Investigation Clinical Network Data Coordinating Center—to harmonize consortium-wide and site-specific efforts for biospecimen collection and analysis. Biospecimen collected are saliva, blood (whole, plasma, serum), urine, stool, and spine tissue (paraspinal muscle, ligamentum flavum, vertebral bone, facet cartilage, disc endplate, annulus fibrosus, or nucleus pulposus). The omics data acquisition and analyses derived from the biospecimen include genomics and epigenetics from DNA, proteomics from protein, transcriptomics from RNA, and microbiomics from 16S rRNA. These analyses contribute to the overarching goal of BACPAC to phenotype cLBP and will guide future efforts for precision medicine treatment.

## Introduction

### BACPAC

In 2019, the National Institutes of Health (NIH) HEAL Initiative formed BACPAC with the primary objective to inform a precision medicine approach to treating chronic low back pain (cLBP). The study is designed to meet the primary objective of investigating an individual’s experience of cLBP through domains of biology, behavior, and biomechanics. To harmonize collection across BACPAC, the Biospecimen Collection and Processing Working Group (WG) has developed standard operating procedures (SOPs) for protocols to be used in all consortium studies. These SOPs detail specimen collection, processing, storage, and analysis across studies and sites both internal and external to BACPAC. Also funded by the HEAL Initiative is the Early Phase Pain Investigation Clinical Network’s Data Coordinating Center (EPPIC-Net DCC) at the New York University (NYU) Center for Biospecimen Research and Development (CBRD), which interfaces with the BACPAC sites to store and distribute the biospecimen and optimize use of biospecimen procedures.

### Chronic Low Back Pain

The causes of cLBP are varied and can be both mechanical and nonmechanical, and can be worsened by risk factors including inactivity, smoking, age, and others [1–3]. Biomarkers have been studied extensively in known contributors to cLBP [4–6] such as intervertebral disc (IVD) degeneration (IDD) [7], modic changes [8–10], and fibromyalgia [11]. The significant association of disparate biomarkers with the causes of cLBP and the response to treatment supports the need for integrative and harmonized “omics” approach that has been developed and implemented by BACPAC.

Several reviews have been published on the significant relationship between serum biomarkers and LBP—most notably, C-reactive protein (CRP), interleukin 6 (IL-6), and tumor necrosis factor (TNF)-alpha [12–20]. In a review by Zhao et al. [21], the authors mapped changes in disc cell types to their implications for IDD. The types of change identified were matrix synthesis, catabolic metabolism, growth factors, and pro-inflammatory cytokines and their receptors. Zhao et al. concluded that mapping cell phenotypes involved in IDD and aging illuminated both causes and potential treatment targets.

However, it has become increasingly clear that biomarkers associated with individual spinal structures and the experience or symptomology of pain, and therefore the treatment of pain, is not homogeneous. In fact, although overlap exists, it is likely that biomarkers of local tissue damage, such as inflammatory and catabolic markers, may be more prominent in some populations, while biomarkers related to the experience of pain, such as neurotransmitters, are more prominent in others [22, 23]. A broad phenotyping strategy that includes biospecimen analysis, as undertaken by BACPAC, better represents the breadth of symptomology and guides a precision medicine treatment approach informed by relevant biological biomarkers. This paper describes the harmonized biospecimen collection, processing, and analyses in BACPAC.

## Biospecimen Collection and Processing

The EPPIC-Net DCC wrote a BACPAC Laboratory Manual to harmonize specimen collection, labeling, processing, cataloging, storage, and shipment to the CBRD; research personnel at all sites received standardized training in collection and processing procedures. The types of biospecimen collected at each of the three sites and their timing are depicted in Table 1. Samples collected consist of whole blood, saliva, urine, stool, and spinal tissue obtained at surgery. A summary is described here while the BACPAC Laboratory Manual, which contains full details of all procedures, is included in Supplemental Data Appendix 1. The time window allowed for spinal tissue collection, processing, and storage at −80°C is 30–60 minutes—a similar time requirement (within an hour) applies for serum, plasma, and saliva collection and storage. Stool collection occurs at home so the time window for collection, processing, and storage is longer. The manufacturer recommended protocol was followed for specimen tubes and is detailed below.

**Table 1.** Timeline of sample collection by MRCs: University of Pittsburgh (PITT); University of California, San Francisco (UCSF); University of Michigan (UMICH)

	Baseline	12 Months	24 Months	36 Months	Surgery*
Whole blood—plasma	PITT	UCSF	UCSF	UCSF	
	UCSF				
	UMICH				
Whole blood—serum	PITT	UCSF	UCSF	UCSF	
	UCSF				
	UMICH				
Whole blood—RNA PAXGene	PITT				
	UCSF				
	UMICH				
Whole blood—DNA PAXGene	UCSF				
Saliva	PITT				
	UCSF				
	UMICH				
Urine	PITT				
Stool	PITT				
	UCSF				
Spine tissue					PITT

* May occur over entirety of enrollment period.

### Inter-site Harmonized Collection and Processing Protocols

#### Blood Samples (Laboratory Manual p. 11–12)

Blood samples are collected at the baseline visit for all sites and at every visit at University of California, San Francisco (UCSF). Collection is done in the morning and is not restricted to fasting. A total of four vials are collected using standard venipuncture technique by trained phlebotomists: whole blood for plasma is collected in one 10 mL K2 EDTA blood draw tube, and aliquoted into cryovials; whole blood for serum is collected in one 10 mL serum separating tube (SST); whole blood for RNA and DNA PaxGene analysis is collected in two 2.5 mL PAXgene Blood RNA tubes.

For plasma, samples must be centrifuged, aliquoted, and frozen within 60 minutes of collection. After centrifugation, plasma is aliquoted into labeled plasma cryovials and immediately stored upright at −80°C until shipping. For serum, the SST tube must be processed after a minimum of 30 minutes to allow a clot to form, and frozen at −80°C. The tube is then centrifuged and aliquoted into labeled cryovials and within the hour frozen at −80°C. For whole blood for RNA and DNA PaxGene analysis, the tubes are stored upright at room temperature for a minimum of 2 hours, frozen at −20°C for 24 hours, and then transferred and stored at −80°C.

#### Saliva samples (laboratory Manual p. 13)

Saliva samples are collected during the baseline visit and are used for DNA extraction. Participants are instructed not to eat, drink, smoke, or chew gum for 30 minutes before giving the sample. Saliva is collected using the OrageneDISCOVER (OGR-500) saliva kit. Participants fill two vials with liquid saliva to the 2 mL line, and the tubes are immediately shaken for 5 seconds. The saliva is aliquoted into labeled cryovials and stored in a −80°C freezer until shipment or analysis.

#### Urine (Laboratory Manual p. 14)

Urine is collected in a sterile urine collection cup; approximately 3.5–4.0 mL are transferred into two 4.5 mL labeled cryovials using a transfer pipette, and frozen at −80°C.

#### Stool (Laboratory Manual p. 15)

During the baseline visit at UCSF and PITT, each participant is offered an optional home stool collection kit, instructions, and shipping envelope. The stool is collected by the participant at home, using a DNA/RNA Shield Fecal collection tube (∼1 g of sample is needed) and shipped to CBRD on cool gel packs. At PITT, participants who return a stool sample also complete the Food Frequency Questionnaire. Once stool samples are received, they are stored at −80°C.

#### Biospecimen Labeling, Storage, and Inventory

At the end of the study, remaining biospecimen are batch shipped frozen on dry ice to the CBRD for long-term storage, except for the stool specimen sent directly from participants. These shipments follow IATA PI 650/UN3373 Regulations for Biological Substance Category B Shipments. At the CBRD, biospecimen are catalogued in LabVantage, a secure network linking biospecimen to corresponding clinical and pathological data. LabVantage allows for sample management without using identifying personal health information (PHI). Each aliquot and tube is labeled with a de-identified barcode that uniquely identifies each biospecimen collected for each participant at each visit across all sites; PHI is maintained at the study sites. The CBRD provides collection sheets to document the samples collected at each visit, aliquots processed and stored at the sites, and the aliquots shipped to CBRD, along with storage details. The CBRD and LabVantage meet all General Lab Protocol and FDA guidelines.

### Site-Specific Collection and Processing Protocols

#### University of Michigan (UMICH)

*Ex Vivo Immune Stimulant.* The TruCulture^®^ system (Rules Based Medicine, Austin, TX) consists of small vacutainers pre-loaded with immune stimulants (e.g., LPS) or control media. Whole blood is drawn via venipuncture into two different 1 mL tubes containing lipopolysaccharide (LPS; 100 ng/mL) or media (NULL). Samples are immediately incubated at 37°C for 24 hours, after which the supernatant is isolated with a valve separator included in the kit. The supernatant is aliquoted into 0.5 mL containers and stored at −80°C for batch analysis.

*Sex Hormones.* Five mL of whole blood are drawn into standard “red top” silicone-coated vacutainers. Whole blood is allowed to clot for 15–30 minutes. Samples are then centrifuged for 10–20 minutes at 1,000–2,000 rcf for isolation of serum. These are subsequently aliquoted into 2 mL cryovials and stored at −80°C for batch analysis of hormones.

#### University of California, San Francisco (UCSF)

Peripheral blood cells are collected at baseline from patients enrolled at UCSF. Following venipuncture, blood collected into EDTA plasma tubes are inverted 8–10 times and soared upright at 4°C for 1–2 hours. Tubes are centrifuged (15 minutes, 1300 rcf, 4°C) and plasma removed without disturbing buffy coat. Entire cell fraction is transferred to a 15 mL conical tube prefilled with 12 mL Ammonium-Chloride-Potassium (ACK) lysing buffer and incubated for 10 minutes on ice or at 4°C until solution becomes translucent. Tubes are gently inverted several times during incubation. Cells are collected by centrifugation (5 minutes, 400 rcf, 4°C) and washed with 12 mL PBS without magnesium and calcium. After centrifugation, cells are dissolved in 2 mL Recovery Freezing media (GIBCO, Cat No. 12648–010) and cryopreserved in 2 × 1 mL cryo tubes. Cells are frozen at −80°C at −1°C/min using Mr. Frosty^TM^. The next day, cells are transferred to vapor phase of liquid nitrogen.

#### University of Pittsburgh (PITT)

At the baseline visit participants are directed to provide a urine sample in a supplied collection cup (30–60 mL). After collection, 3.5–4.0 mL is aliquoted into two 4.5 mL cryovials. The cryovials are stored in a −80°C freezer until shipment.

Tissue samples are collected from a subset of the participants—those who are enrolled and undergo spinal procedure during the study timeframe (Laboratory Manual p.17–18). Tissue samples will not be collected from a single surgical level because it will be dependent on the surgery. The spinal level will be recorded. The following tissues will be collected: paraspinal muscle, ligamentum flavum, vertebral bone, facet cartilage, disc endplate, annulus fibrosus, or nucleus pulposus. At the time of surgical procedure spinal tissue is placed into a sterile cup with 10 mL of PBS to remove blood product from the tissue. The tissue is removed from the cup and blotted on sterile gauze to remove excess liquid. Tissue is immediately placed into a labeled 5 mL cryotube and immersed in a bucket of wet ice. When the tissue arrives at the laboratory, within 30–60 minutes, 100 mg (5 mm cubes) aliquots are cut using a sterile blade. Each aliquot is placed into a 2 mL cryovial and the tube is weighed to calculate tissue mass. The tube is submerged in liquid nitrogen to flash freeze for −80°C storage.

## Planned Biomarker Data Acquisition and Analyses

### Biomarkers

**Figure 1.**
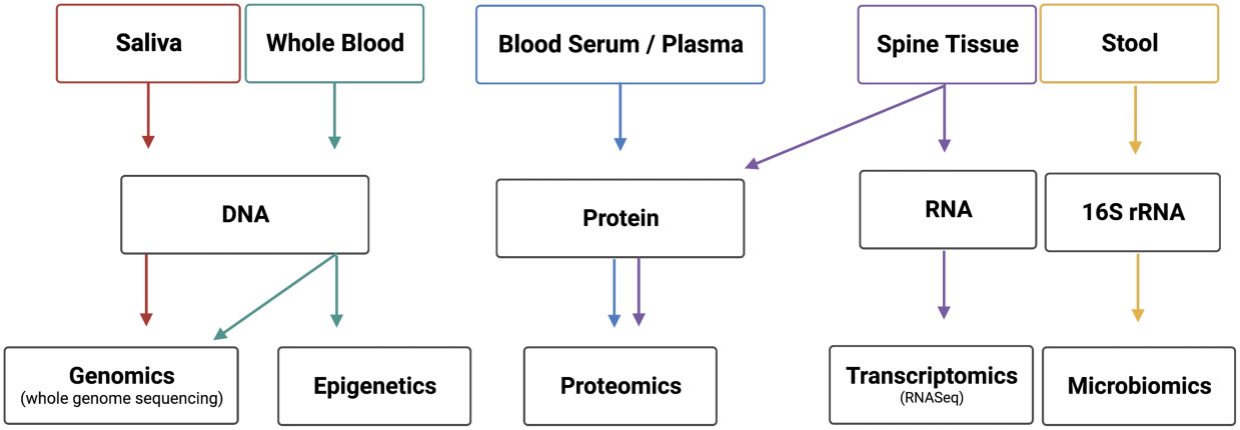
Summary schematic of biospecimen and omics analyses.

As discussed above, there are limitations in previous studies, which BACPAC hopes to address with the collection and analysis described herein of a network of biological biomarkers with corresponding behavioral and biomechanical biomarkers. The collection of some samples was decided based on the ability to conduct multiple analyses—that is, DNA collection in both saliva and blood allows for genetic, epigenetics, and microbiomics. Similarly, the decision was made to collect both blood plasma and serum to maximize the analysis potential from each participant. A summary schematic of the biospecimen and planned analysis are presented in Figure 1 and Table 2 and discussed in greater detail below.

**Table 2.** Summary of analysis

Biological Sample	Amount	Analysis
Blood—plasma	1 × 10 mL tube	ELISA
		MS-based proteomics
Blood—serum	1 × 10 mL tube	ELISA
		MS-based proteomics
Blood—RNA	2 × 2.5 mL tubes	RNAseq
Saliva	2 × 2 mL	Whole genome sequencing
Urine	2 × 4.5 mL	ELISA
Spinal tissue	200–1000 mg	RNASeq
Stool	520 ± 101 mg	Bacterial RNA

* Spine tissue samples can include ligamentum flavum, facet joint cartilage, cartilaginous endplate, and intervertebral disc nucleus pulposus and annulus fibrosus.

MS = Mass spectrometry; ELISA = enzyme-linked immunosorbent assay.

### Proteomics

The complexity of cLBP and the lack of universal biomarkers have slowed hypothesis-driven reductionist approaches to identifying the molecular mechanism driving cLBP and treatment response in patients. To overcome the current barriers of cLBP biomarker studies, we propose to perform broad unbiased proteomic studies to vastly increase the depth of biological information to identify unique profiles of biomarkers describing different cLBP phenotypes. First, our proposed serum and proteomics will comprehensively measure and analyze a large number of proteins increasing the likelihood of unique insight.

Advances in proteomic methods now permit thousands of proteins to be profiled from trace amounts of plasma. Mass spectrometry- (MS) based proteomics has the unique advantage of being unbiased and quantitative. Previous reported work suggest that large-scale proteomics analysis is a promising way of discovering novel biomarkers that could substantially improve the prediction of Alzheimer’s disease [24, 25], ischemic stroke [26, 27], and cancer [28]. Furthermore, the importance of sufficiently large discovery cohorts has become increasingly evident. MS is also a good technology for qualifying putative biomarkers in large numbers of clinical samples. Specifically, the combination of selective reaction monitoring and stable isotope labeled standards permit the development of highly multiplexed clinical assays that can quantify hundreds of proteins without the use of antibody reagents.

Label-free differential MS (dMS) was previously established as an unbiased, robust approach to identify differences in protein expression in in vitro experiments, preclinical species, and humans. A key advantage of a dMS approach is that it supports large multi-level study designs that compare protein expression across multiple timepoints and/or treatment conditions. High-resolution Fourier transform MS is used to profile complex samples derived from serum of cLBP patients. The exquisite mass resolution afforded by modern high-resolution MS are capable of separating ions that differ by as little as 0.005 m/z units and resulting in excess of 100,000 molecular features per profile. Sophisticated software is used to extract m/z, r.t., and relative abundance values for each of the features extracted from the LC-MS data. Features that exhibit statistically significant differences in abundance are selected by ANOVA analysis and identified by tandem MS (MS/MS). Briefly, candidate features are subjected to collision-induced dissociation or electron transfer dissociation to generate high-resolution MS/MS that can be used for database searching or de novo sequence identification. Candidate LBP biomarkers that are identified in these discovery proteomics experiments will be prioritized for follow-up in qualification studies.

A key step for any proteomics experiment is the development and optimization of the biochemical sample preparation methods that are custom tailored to a particular biofluid type. Serum—which is challenging to analyze due to the presence of albumin and other high abundance immunoglobulins protein—is processed using a commercialized immune-depletion kit that enables the detection of hundreds of proteins from a small sample aliquot. To remove these abundant proteins, immunoaffinity techniques are used that enable the removal of the most abundant proteins, thus enhancing the detection of lower-abundance proteins in both discovery and targeted proteomic analyses. Experiments using pooled samples (technical replicates) are conducted to characterize the effect of samples storage (freeze/thaw), sample processing, and LC-MS/MS analysis.

### Transcriptomics

The transcriptome refers to all RNA transcripts in a biological sample (e.g., blood, cells), providing insights into the molecular components and processes of cells and tissues at normal and pathologic states. Historically, transcriptomic analyses have focused on mRNA that code for proteins, but now often include additional non-codding RNA sub-types. A recent study identified differential gene expression in blood between controls and cLBP patients, as well as between acute LBP and cLBP groups [29, 30]. Both comparisons showed enriched expression of genes in the extended major histocompatibility complex (MHC) locus, including genes in MHC class I and class II of cLBP patients, and many of these genes have been identified in previous studies of chronic pain and inflammatory conditions characterized by chronic pain [29]. Additionally, upregulated gene expression has been observed in cLBP characterized by neuropathic pain when compared to controls, particularly the tissue inhibitor of matrix metalloproteinase-1 (TIMP1) gene—as physiologic levels of TIMP1 were also higher in neuropathic cLBP versus inflammatory cLBP, these findings suggest a role for transcriptomic analysis in identifying cLBP subtypes [31]. Finally, a small feasibility study suggested that an increase in antisense transcripts of genes associated with pain states after a successful yoga intervention, lending credibility to the use of transcriptomics to identify treatment response in cLBP [32].

RNA is isolated from the PAXgene™ RNA Tubes. For each sample, RNA libraries are prepared from 500 ng of high-quality RNA (RIN ≧ 7) using the KAPA mRNA HyperPrep Kit (Kapa Biosystems). The cDNA libraries are pooled at a final concentration of 1.8 pM and then sequenced using NovaSeq6000 platform (Illumina) to an average of 50M reads. Differential expression is quantified using DESeq2—comparing low versus high pain intensity and disability in participants. Read counts are normalized across all samples and significant differentially expressed genes are determined by adjusted *P* values with a threshold of 0.05. Using RNA sequencing we can determine how baseline transcriptomics correlate with the severity of cLBP and changes in LBP symptoms with time, or with or without treatments, in this participant population. RNA quality will be determined by RNA Integrity Number (RIN), which is required to be within 7–10 to be subjected to RNA-Seq. Hierarchical clustering and principal component analysis (PCA) show unbiased population-specific clustering and provide a systematic view of gene differential expression of the RNAseq data.

### Microbiomics

The gut microbiota is a signaling hub that integrates environmental inputs with genetic signals to regulate host immunity, inflammation, and response to infection [33]. This microbial community starts developing at birth, contains 150-fold more genes than our human genome [34], protects the host from invading pathogens, and prevents immune responses to harmless commensal microbes [35]. There is growing evidence that gut bacteria dysbiosis associates with numerous disease susceptibilities, such as IBD, diabetes, multiple sclerosis, and cardiovascular disease [36, 37]. Despite these emerging associations, the impact of gut microbiota on musculoskeletal health is largely unexplored [38].

The gut microbiome could modulate multiple biopsychosocial factors that are known to be associated with cLBP. Microbiome-host interactions modulate inflammatory cytokine production capacity. Thaiss et al. categorized three types of interactions that are crucial for preserving tissue homeostasis and that contribute to microbiome-mediated disease phenotypes [33]. Of potential relevance to cLBP, microbial products can serve as perpetual stimuli of chronic immune responses that contribute to persistent inflammation. Specifically, gut commensals calibrate innate and adaptive immune responses and impact the activation threshold for pathogenic stimulations by producing metabolites that modulate cytokine production and induce expansion of regulatory T cells. For example, Schirmer et al. showed that inter-individual variation in TNF-α and interferon (IFN-γ production is linked to specific byproducts of microbial metabolism [39]. The presence of specific bacteria is also required for T helper cell 17 (Th17) cell differentiation in the small intestine and spinal cord [40, 41]. Since immune cell infiltration into spinal tissues is a central driver of the intra-spinal pro-inflammatory cascade and is thought to mediate patient symptoms [42], understanding the role of the microbiome in the systemic inflammation that accompanies cLBP is paramount.

Several studies using preclinical models have demonstrated the association between gut microbial composition and pain including neuropathic, inflammatory, and visceral pain [43, 44]. For example, oxaliplatin-induced neuropathic pain was reduced in germ-free mice and antibiotic-treated mice while restoring the microbiota of germ-free mice abolished the effects [45]. The gut microbiome could impact certain patients’ abilities to cope with chronic pain and to adhere to treatment interventions by producing metabolites that regulate moods such as motivation, anxiety and depression [46, 47]. For example, gut bacteria manufacture about 95% of the body’s supply of the neurotransmitter serotonin from the essential amino acid tryptophan, which influences both mood and GI activity [48].

Microbiome data are commonly obtained in three forms: 1) 16S rRNA gene sequence surveys that provide a view of microbiome bacterial membership; 2) metagenomic data used to portray genetic functional potential; and 3) meta-transcriptomic data to describe active gene expression. BACPAC sites that collect patient stool samples (UCSF, PITT) focus primarily on 16S rRNA gene surveys because they are economical for large projects and because 16S rRNA gene sequence data provide a relatively unbiased characterization of bacterial and archaeal diversity [49]. For 16S rRNA sequencing, DNA is extracted from stool samples using a modified cetyltrimethylammonium bromide buffer extraction protocol [50].

One proposed method of data analysis focuses primarily on measuring bacterial diversity. A table of amplicon sequence variants (ASVs) and assigned taxonomy are generated using the NG-Tax 2.0 pipeline [51]. ASVs are discarded if determined to be chimeric, nonbacterial origin, common contaminants observed in >50% of extraction controls, or have read counts less than 0.001% of the total read count. Sample read numbers are representatively rarefied to 20,000 reads as a means of normalization [52]. α-Diversity, a within-sample diversity measure and β-Diversity, a between-samples compositional dissimilarity measure, is calculated.

### Genomics

In a recent meta-analysis, three genetic loci were identified which have significant association with cLBP; in this study the DCC gene was found to reside proximal to one of these loci that encodes a netrin 1 receptor, a transmembrane protein member of the immunoglobulin superfamily of cell adhesion molecules, which mediates axon guidance of neuronal growth cones that could potentiate pain [53]. There are a number of genetic single nucleotide polymorphisms (SNPs) associated with LBP and IDD, including those within the cytokine and metalloproteinase gene families. However, due to cost and feasibility issues, most studies to date on linking genetic makeup to LBP are still limited on identifying genetic variants (e.g., SNPs) without considering all different types of biomarkers in the same human subjects. Compared to the typical selective genetic variants shown in Table 3, our whole genome sequencing provides depth to already existing genomic data.

**Table 3.** Genetic SNPs associated with LBP and IDD

Gene Class	Gene	Refs
Cytokines	IL-1a, IL-1b, IL-4, IL-6, IL-10	[81–85]
Growth factors	TGFb, BDNF, VEGF, GDF5	[86–88]
Matrix	ACAN, COL1A1, COL9A1	[89–91]
Metalloproteinases	MMP-1, MMP-3, MMP-9, ADAMTS-4	[92–95]
Serotonergic and adrenergic pathways	HTR1A, COMT, ADRB2	[96–99]
Ion channels	KCNJ6, TRPV1	[100, 101]
Genetic loci	(Nearest genes) SOX5, DIS3L2, DCC, CCDC26/GSDMC	[48, 53]
Miscellaneous	CYP2D6, VDR, CASP9, FAAH, IFN-a, IFN-y	[101–105]

Genome-wide sequencing of 1,000 cLBP participants is performed using collected saliva and whole blood samples. Genomic DNA is isolated from saliva or whole blood and DNA libraries are prepared. 500 ng of genomic DNA goes through fragmentation, enzymatic end-repair and A-tailing, ligation, followed by quality check. Libraries with an average size of 450 bp (range: 300-600bp) are quantified by qPCR, normalized, and pooled per manufacturer protocol (Illumina). Finally, sequencing is performed to an average target depth of 30-40x coverage (guaranteed >20×).

Genome-wide association analyses is performed using logistic regression models with additive genetic effects, between control (NIH database) and cLBP cohorts [53, 54]. Data harmonization and quality control is conducted using the EasyQC software package in the R statistical environment. The meta-analysis of autosomal SNPs is performed with METAL, (http://csg.sph.umich.edu/abecasis/metal/), using the LDsr intercept as a correction factor [55], to combine results from all cohorts. Q-Q and Manhattan plots are generated with R to present the findings.

### Epigenetics

Epigenetics is defined as “molecular factors and processes around DNA that regulate genome activity, independent of DNA sequencing, and are mitotically stable.” [56] Twin studies indicate that 30–75% of LBP is heritable [57–59]. Environmental factors such as high psychological and physical stress associate with cLBP [60, 61]. Epigenetic mechanisms can link environmental factors to heritable genetic expression and hence play an important role in cLBP. DNA methylation is a major epigenetic process that describes the methylation of cystein in CpG dinucleotides. Typically, DNA methylation is present in mammals only at CpG dinucleotides, which are methylated by 70–85%. In contrast, CpG islands are mainly unmethylated to remain active [62]. About 70% of promoters in human contain CpG islands [63]. Genome-wide DNA methylation analysis is one of the most common epigenetic processes analyzed for genome characterization [56].

The invention of bisulfite sequencing in 2009 opened the possibility for genome-wide methylation analysis. IVD and ligamentum flavum show degeneration-dependent differential DNA methylation of genes and promoters relevant for the pathophysiology [64–66]. For example, methylation of the SPARC promoter affects chronic pain in humans and animals [66]. Epigenetic changes in blood cells as biomarker or as phenotypic trait of cLBP patients has only started to be investigated in the past few years. In 2013, a whole blood methylation study of 38 individuals from the UK Twin cohort identified hypermethylated CpG islands in the promoter of the PARK2 gene to be correlated with IDD [67]. Aroke et al. compared DNA methylation of 50 cLBP patients and 50 healthy individuals and found that most differentially methylated genes were located in CpG islands and promoter regions for genes involved in immune signaling, endochondral ossification, G-protein coupled transmissions, pain processing, and neuronal differentiation [68, 69]. Methylation of genes related to neuronal differentiation, growth, and plasticity may also be involved in impaired conditional pain modulation in cLBP patients [70]. Studies from 2021 suggest a role of lymphocytes in cLBP. Eller et al. found that increased IL-2 expression correlated with lower global DNA methylation, suggesting that epigenetic changes may be linked to activation of lymphocytes in cLBP [71]. Grégoire et al. compared DNA methylation in circulating T cells in cLBP patients and healthy controls and identified CpG sites that are able to categorize the pain status [72]. Together these studies indicate that epigenetic regulation is important in the pathophysiology of cLBP.

When DNA is treated with sodium bisulfite, unmethylated cysteines are converted to uracil while methylated cysteines remain unchanged. After sequencing of the bisulfite treated DNA, the reads can be mapped to the original genome to identify cysteine-to-uracil changes at a single nucleotide level. Whole-genome bisulfite sequencing (WGBS) and reduced representation bisulfite sequencing (RRBS) can be used. RRBS uses methylation insensitive restriction enzymes to cleave the DNA at CpG dinucleotides to enriches for promoter regions and hence allow greater read depth compared to WGBS.

UCSF uses RRBS to investigate DNA methylation in whole blood of cLBP patients. Briefly, peripheral blood is collected in PAXGene DNA tubes and purified using a robotic workstation to minimize handling variations. DNA is cleaved with the restriction enzyme MspI, 5′CG overhangs repaired, A-tails added, and methyl adapter ligated. DNA fragments of 20–120 bp and of 120–220 bp length are isolated and cysteines converted to uracil using bisulfite. The bisulfite converted DNA is barcoded, amplified, and sequenced with Novaseq S1 using 20 million paired-end reads per sample with at least 150 base pairs per reads. Sequencing quality is assessed, reads aligned to the human reference genome, and methylated cysteins identified. Genomic features (i.e., adjacent genes, adjacent cis-regulated regions) of methylated regions are annotated.

### Immune Cell Analyses

Chronic pain conditions can affect the phenotypic profile of circulating immune cells [73]. In cLBP patients, the number of circulating natural killer (NK) cells are reduced as well as the ratio of Th17 to regulatory T cells (Treg) [74, 75]. This indicates an anti-inflammatory T cell shift in cLBP patients. The changes in immune cell ratios can be related to pain mechanisms and to local tissue reactions. For example, disc herniation, disc degeneration, and Modic changes cause local activation and proliferation of different subsets of immune cells [42, 76, 77]. In the case of herniation, exposure of the immune privileged disc to the immune system recruits CD4^+^ cells [77]. Increased CD4^+^/CD8^+^ ratio in LBP patients with herniated discs can be detected in peripheral blood [78]. In the bone marrow that is affected by Modic changes, neutrophilic and lymphocytic infiltrates were described [76, 79]. Yet, alterations in circulating cell populations in Modic change patients have not been reported. Integrated analysis of pain measures with radiologic and flow cytometric analysis may help clarify if changes in the proportion of circulating immune cells are related to local tissue changes or the LBP experience.

Spectral flow cytometry will be used for immune-profiling of peripheral blood. For staining, OMIP-69, an optimized immunophenotyping panel will be used [80]. This panel contains 40 markers to characterize CD4 T cells, CD8 T cells, Tregs, γδ T cells, NKT-like cells, B cells, NK cells, monocytes, and dendritic cells.

## Conclusions

These efforts represent a broad, comprehensive approach to biospecimen collection that will facilitate novel biological biomarker evaluation. As biospecimen handling, processing, and analysis procedures are harmonized, larger datasets are created as a benefit to BACPAC. In addition, given the vast and varied data being collected throughout BACPAC on the same participants, more comprehensive analyses can be accomplished considering all contributors to the complex condition of cLBP. This approach has a higher likelihood of yielding clinically relevant targets, both for prognosis and treatment. Finally, this work will produce an invaluable biorepository that can later be queried as novel hypotheses are developed, with the goal of improving outcomes for this common and costly condition.

## Supplementary Material

pnac197_Supplementary_Data
