# Supplementary material for: Protocol for Biospecimen Collection and Analysis Within the BACPAC Research Program
Source: Pain Med. 2022 Dec 16;24(Suppl 1):S71–80. doi: 10.1093/pm/pnac197 (PMC10403310; doi:10.1093/pm/pnac197)
Supplement: pnac197_Supplementary_Data [file pnac197_supplementary_data.pdf]

NYU Center for Biospecimen Research and Development (CBRD)

EPPIC-Net: BACPAC

CONFIDENTIAL

LABORATORY MANUAL

VERSION: 1.6

DATE: 11 Apr 2022

PRIMARY INVESTIGATORS:
EVA PETKOVA, PhD, NEW YORK UNIVERSITY SCHOOL OF MEDICINE
ANDREA B. TROXEL, ScD, NEW YORK UNIVERSITY SCHOOL OF MEDICINE

TABLE OF CONTENTS

GENERAL INFORMATION
- Laboratory Manual Version . . . . Page 3
- Contact Information . . . . Page 4
- Weekend and Holiday Considerations. . . . . Page 5
- Label Re-Supply Orders. . . . . Page 6

BIOSPECIMEN MANAGEMENT
- Patient-Specific Label Envelopes. . . . . Page 7
- Visit Schedule. . . . . Page 8-9

SPECIMEN PROCESSING PROCEDURES
- Labeling Procedure. . . . . Page 10
- Blood . . . . Page 11-12
- Saliva. . . . . .Page 13
- Urine. . . . . .Page 14
- Stool. . . . . .Page 15-16
- Spinal Tissue . . . . Page 17-18

SPECIMEN REQUISITION FORMS
- Patient Specific Collection Sheets . . . . Page 19
- Biofluids Requisition Form . . . . Page 20
- Spinal Tissue Requisition Form . . . . Page 21
- Stool Requisition Form . . . . Page 22

SHIPPING SPECIMENS TO CBRD CENTRAL LABORATORY
- Packaging Instructions . . . . .Page 23-24

LABORATORY MANUAL VERSION

Version	Revision Dates
V. 1.0	21 April 2020
V. 1.1	6 May 2020
V. 1.2	21 May 2020
V. 1.3	28 May 2020
V. 1.4	15 Jul 2020
V. 1.5	15 Oct 2021
V. 1.6	11 Apr 2022

CONTACT INFORMATION

EPPIC-Net DCC Principal Investigators Information:

Name	Email/Phone
Eva Petkova, PhD	Eva.petkova@nyulangone.org<br>+1 646-501-3653
Andrea B. Troxel, ScD	Andrea.troxel@nyulangone.org<br>+1 646-501-3654

CBRD CENTRAL LABORATORY Contact Information:

Name	Email/Phone
Estefania Toro Montoya	Estefania.ToroMontoya@nyulangone.org<br>+1 646-501-4268
Bernice Pham	Bernice.pham@nyulangone.org<br>+1 646-501-4268
Sandra Mendoza	Sandra.Mendoza@nyulangone.org<br>+1 646-501-4268

All specimens (except safety lab) collected on this study will be shipped to CBRD.

Center for Biospecimen Research and Development (CBRD)
NYU Langone Medical Center
Sandra Mendoza
550 1st Avenue, Medical Science Building, room 238
New York, New York 10016

eppic-netbiorepository@nyulangone.org
PH: 1 (646) 501-4268
Fax: 1 (646) 501-4579

WEEKEND AND HOLIDAY CONSIDERATIONS

LOCAL COURIER SERVICE (PICKUP AND DELIVERY) MAY BE LIMITED PRIOR TO, DURING AND FOLLOWING OBSERVED HOLIDAYS IN THE U.S.

PLEASE DO NOT SCHEDULE A SHIPMENT THE DAY BEFORE A HOLIDAY.

CBRD Central Laboratory at NYU Langone Medical Center will be closed for the following holidays and unable to accept shipment of specimens:

NYULMC HOLIDAY SCHEDULE
New Year's Day
Martin Luther King Jr. Day
President's Day
Memorial Day
Independence Day
Labor Day
Thanksgiving Day
Christmas Day

FRIDAY AND WEEKEND SHIPMENTS TO NYU ARE NOT PERMITTED. CBRD Central Laboratory at NYULMC Health is CLOSED Saturday and Sunday.

LABELS RE-SUPPLY ORDERS

1. To request patient specific labels for draw blood tubes or cryovials, login into EPPIC-Net DCC LabVantage WebAccess portal and fill out labels order.
2. If labels are urgently required - send an email to eppic-netbiorepository@nyulangone.org.
3. Allow a minimum of 5 business days to process any site labels requests (including initial and resupply orders). Delivery transit depends on site location, please allow transit time (4-7 days) in addition to the 5 business for delivery at site.

BIOSPECIMEN MANAGEMENT

PATIENT-SPECIFIC ENVELOPES FOR PRE-PRINTED LABELS

- **DRAW TUBE AND ALIQUOT CRYOVIAL LABELS WILL BE PROVIDED IN SITE AND PATIENT-SPECIFIC ENVELOPES**
- **ALL LABELS MUST BE COMPLETED BY HAND WITH THE DATE OF COLLECTION, USE A SHARPIE PEN**
- **LABELS ARE SUPPLIED FOR MAINTENANCE OF BIOSPECIMEN INVENTORY-ALL LABELS ARE PRE-PRINTED WITH THE SITE NUMBER, VISIT TIME POINT, AND PATIENT ID #-DO NOT EXCHANGE LABELS BETWEEN PATIENTS!**
- **REQUISITION FORM MUST FILLED OUT WITH PATIENT ID, VISIT TYPE, SITE AND THE DATE OF COLLECTION**
- **PATIENT-SPECIFIC LABELS CAN BE ORDERED THROUGH the EPPIC-Net DCC LabVantage WebAccess Portal**

DATE OF COLLECTION must be completed on labels of any tube being sent to CBRD Central Laboratory

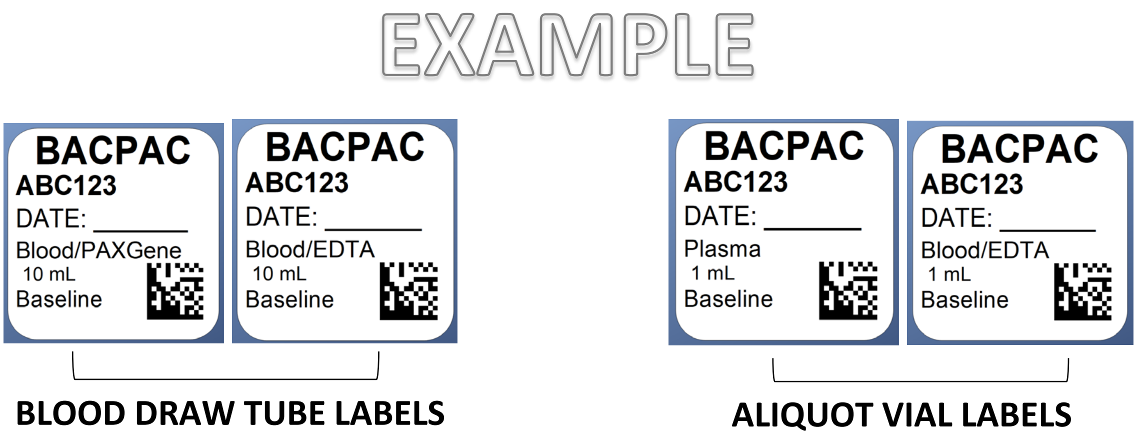

BIOSPECIMEN MANAGEMENT

VISIT SCHEDULE: UCSF, UCD, UCI, UCSD

Specimen Type	Baseline	12 months	24 months	36 months	Surgery*
**BIOFLUIDS**					
Whole Blood: Plasma	X	X	X	X	
Whole Blood: Serum	X	X	X	X	
Whole Blood: RNA PaxGene	X				
Saliva	X				

- TBD Collection to be determine
- * No Samples Collected at Surgery

VISIT SCHEDULE: UMICH

Specimen Type	Baseline	12 months	24 months	36 months	Surgery*
**BIOFLUIDS**					
Whole Blood: Serum	X				
Whole Blood: RNA PaxGene	X				
Saliva	X				
Urine	TBD				

- TBD Collection to be determine
- * No Samples Collected at 12, 24, 36 months, surgery

VISIT SCHEDULE: U. Pittsburg

Specimen Type	Baseline	12 months	24 months	36 months	Surgery*
**BIOFLUIDS**					
Whole Blood: Serum	X				
Whole Blood: Plasma	X				
Saliva	X				
Urine	TBD				
Stool	TBD				
**SPINAL TISSUES***					
Paraspinal muscle					X
Ligmentum flavum					X
Vertebral bone					X
Facet cartilage					X
Disc endplate					X
Disc annulus fibrosus					X
Disc nucleus pulposus					X

- * Collect if patient goes on to surgery
- TBD Collection to be determine

SPECIMEN PROCESSING PROCEDURES

LABELING PROCEDURE

The correct way to label the tube is to place the label such that the words are parallel to the direction of the tube (vertically). This way, the information can be read without needing to rotate the tube.

The incorrect way to label is placing the label with the words wrapping around the tube (horizontally). See images below:

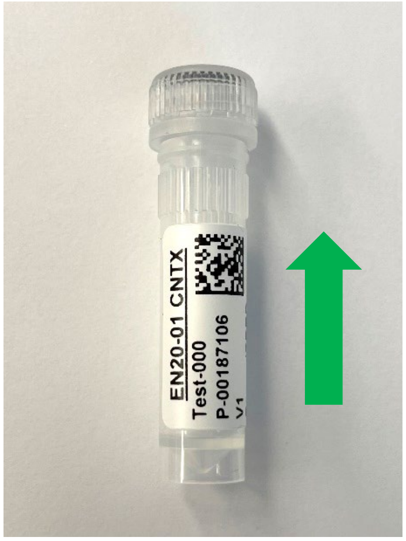

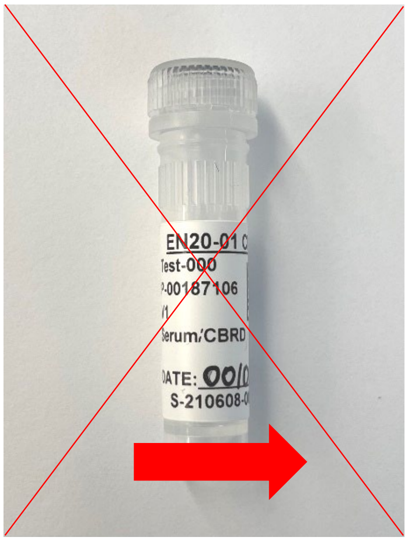

CORRECT **INCORRECT**

SPECIMEN PROCESSING PROCEDURES: BACPAC

Tubes requiring centrifugation must rest on the bottom of centrifuge bucket, otherwise cap from collection tube may loosen during spin.

Please use appropriate calculations for RPM conversion if your centrifuge requires RPM instead of RCF (g force). This calculation must consider the Radius of your centrifuge.

ALL PROCESSING SHOULD OCCUR WITHIN 1 HOUR OF BLOOD COLLECTION

Patients must fast PRIOR blood draws.

BLOOD-PLASMA

Draw blood 10 mL into 1, **EDTA Lavender tube** using standard venipuncture techniques

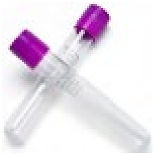

1. Mix the blood by gently inverting tube 8-10 times.
2. Keep the tube on ice until ready to centrifuge; centrifuge within 30-60 minutes after blood collection.
3. Centrifuge for 10 minutes at 1300 rcf, at 4°C.
4. Separate PLASMA from blood sample within 20 minutes of the end of centrifugation.
5. Fully complete the labels on cryovials marked "PLASMA". Label aliquots in numerical order, following the S-ID #'s on the bottom of the label.
6. Use a disposable transfer pipette to transfer the plasma without disturbing the whiteish buffy or red blood cells layers into a 15cc conical tube. Gently mix the plasma with the transfer pipette.
7. Aliquot into the appropriately labelled **blue top**, PLASMA cryovials, 0.5 mL PLASMA PER CRYOVIAL, 10 cryovials per blood tube drawn.
8. Discard tube after obtaining plasma aliquots.
9. Immediately (within 1 hour) freeze and store the samples upright at -80°C until shipment to CBRD Central laboratory.
10. Complete the Requisition Form associated with the visit.
11. Ship frozen on Dry ice according to Batch shipment schedule using Dry ice shipper with Shipping manifest.
12. Send shipment notification to eppic-netbiorepository@nyulangone.org and log shipment in the EPPIC-Net Portal, Biorepository page, LabVantage link.

WHOLE BLOOD RNA

Draw blood into 2, **PAXgene Whole blood RNA tube** (2.5 mL capacity) using standard venipuncture techniques

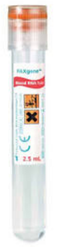

1. Mix the blood by gently inverting tube 8-10 times.
2. Fully complete the labels on the tubes marked "Blood/PGX/RNA".
3. Store PaxGene tubes upright at room temperature for 2 hours.
4. Freeze in wire or plastic rack at -20°C for 24 hours-**NOT STYROFOAM**, tube may crack.
5. Transfer the frozen two samples to a -80°C freezer, store them upright.
6. Complete the Requisition Form associated with the visit.
7. Ship frozen the "PGx RNA" to CBRD on Dry ice according to Batch shipment schedule using Dry ice shipper with Shipping manifest.
8. Keep at -80°C the other "PGx RNA" at the site until requested for analysis.
9. Send shipment notification to eppic-netbiorepository@nyulangone.org and log shipment in the EPPIC-Net Portal, Biorepository page, LabVantage link.

BLOOD-SERUM

Draw blood 10 mL into 1, **Serum-separating tube (SST)** using standard venipuncture techniques

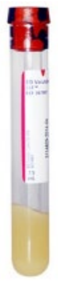

1. Mix the blood by gently inverting tube 8-10 times.
2. In order to obtain serum of high quality, keep tube upright at room temperature for 30 minutes minimum to 60 minutes maximum to allow a clot to form.
3. Centrifuge the blood sample at the end of the clotting time (30-60 minutes) in a horizontal rotor (swing-out head) for 15 minutes at 1300 rcf, at 4°C.
4. Fully complete the labels on cryovials marked "Serum"; 10 each.
5. Use a disposable transfer pipette to transfer the serum into a 15cc conical tube. Gently mix the serum with the pipette.
6. Aliquot all of the serum into the appropriately labelled **red top**, SERUM cryovials, 0.5 mL SERUM PER CRYOVIAL (about 10-14 cryovials). Record number of cryovials used.
7. Immediately (within 1 hour) freeze and store the samples upright at -80°C until shipment to CBRD Central laboratory.
8. Complete the Requisition Form associated with the visit.
9. Ship frozen on Dry ice according to Batch shipment schedule using Dry ice shipper with Shipping manifest.
10. Send shipment notification to eppic-netbiorepository@nyulangone.org and log shipment in the EPPIC-Net Portal, Biorepository page, LabVantage link.

SALIVA

Collect saliva with the **Oragene•DISCOVER** OGR-500 Saliva kit, **2 kits**

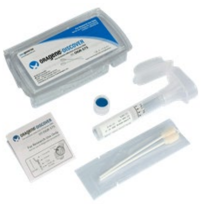

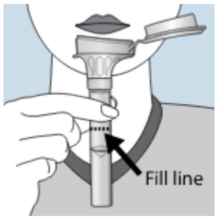

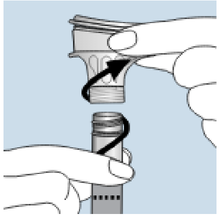

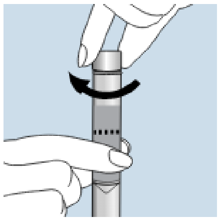

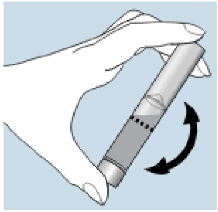

1. Inform the patient on the following collection precautions:
    a. Do **NOT** eat, drink, smoke, or chew gum for 30 minutes before giving saliva sample.
    b. Do **NOT** remove the plastic film from the funnel lid.
    c. Do **NOT** spit above the top line on the tube.
    d. Most people take between 2-5 minutes to deliver the saliva sample following the steps below (3-6)
2. Fully complete the barcoded labels on 6, cryovials marked "Saliva”.
3. Spit a SMALL amount of saliva into the funnel until the amount of liquid saliva (not bubbles) reaches the fill line, 2 mL/vial.
4. Hold the tube upright with one hand. Close the funnel lid with the other hand by firmly pushing the lid until you hear a loud click. The liquid in the lid will be released into the tube to mix with the saliva. Make sure that the lid is closed tightly.
5. Hold the tube upright. Unscrew the funnel from the tube.
6. Use the small caps to close the tubes tightly.
7. Shake the capped tube for 5 seconds. Discard or recycle the funnel.
8. With a transfer pipette, aliquot the saliva specimen from each collection tube, equal volumes, into two cryovials. Cap the tubes tightly.
9. Freeze and store the four samples upright at -80°C.
10. The “SALIVA” samples will follow DNA extraction at the site when requested.
11. Ship to CBRD one frozen “Saliva” aliquot on Dry ice according to Batch shipment schedule using Dry ice shipper with Completed Requisition Form associated with the visit.
12. Send shipment notification to eppic-netbiorepository@nyulangone.org and log shipment in the EPPIC-Net Portal, Biorepository page, LabVantage link.

URINE

Instruct patients to urinate in supplied collection cup

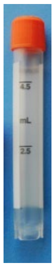

1. Fill out the patient ID and date on the labels for the supplied 4.5 mL cryovials.
2. Collect urine specimen into the provided urine cap.
3. Transfer approximately 3.5-4.0 mL into the two 4.5 mL cryovials marked “Urine” using transfer pipette. Do not fill the tube as the top might pop out once the specimen freezes.
4. Freeze and store at -80°C until shipment to CBRD Central Laboratory.
5. Ship frozen on Dry ice according to Batch shipment schedule using Dry ice shipper with Shipping manifest.
6. Send shipment notification to eppic-netbiorepository@nyulangone.org and log shipment in the EPPIC-Net Portal, Biorepository page, LabVantage link.

Center for
Medicine & the Microbiome
University of Pittsburgh & UPMC

BST E1140
Starzl Biomedical Science Tower
200 Lothrop Street
Pittsburgh, PA 15213

Give the box containing all supplies, instructions, and shipping label for patients to return the samples to the clinic (or ship them to the CBRD central bank). Please check the expiration date of the kit and collection tube **BEFORE** using stool kit for sample collection.

Instruct patients to collect stool by using the **Zymo** kit and put it in the **DNA/RNA Shield Fecal Collection Tube**.

A. STOOL PACKAGING INSTRUCTIONS: Dropping off specimens at the clinic

1. Write the date/time of your stool collection onto the collection tube and stool requisition form.
2. Adhere the paper feces catcher onto the back of the toilet seat (see diagram on paper for further instructions). We have provided you with 2 paper feces catchers in case one rips.
    a. Do not urinate onto the Feces Catcher. If possible, urinate before you adhere the paper catcher.
3. Have your bowel movement onto the paper catcher.
    a. Note: to avoid contamination, do not collect any stool that has fallen into the toilet water.
4. Put on the nitrile gloves included with your kit.
5. Unscrew the collection tube cap & use the spoon to scoop 1 spoonful of feces from your sample.
    a. Please be careful not to spill any of the pre-filled contents of the tube while unscrewing the cap.

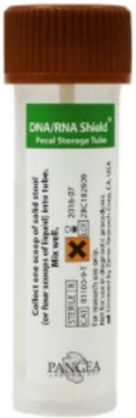

6. Place the sample in the collection tube. Do not touch the scoop or sample that goes into the collection tube.
7. Tighten the cap & shake to mix the contents thoroughly (invert 10 times) to create a suspension.
    a. Note: some fecal material may be difficult to re-suspend. As long as the material is suspended, the sample is stabilized.
    b. Foaming/frothing during shaking is normal.
8. Place sealed collection tube into white Aqui-Pak absorbent pouch (tube separator) and then into the 95kPa specimen transport bag. Seal bag and place completed stool requisition form in the back sleeve of the transport bag.
9. Loosen the paper feces catcher on both ends & squeeze the ends together.
10. Drop the feces catcher in the toilet bowl & wait a moment until the paper is soft & can easily be flushed down the toilet.
11. Dispose of gloves and thoroughly wash your hands.
12. Place sealed specimen bag into the aluminum pouch with the gel pack. Pack aluminum pouch into white shipping box provided in kit and adhere the FedEx airway bill to the outside of the box. Mail this out via FedEx the same day sample is collected.
13. Please visit this website if you would like to watch an instructional video: https://goo.gl/THfEsS

B. STOOL PACKAGING INSTRUCTIONS: Dropping off specimens at the clinic

1. Place the gel pack in the bottom of the aluminum envelope provided.
2. Place the sealed collection bag on top of the gel pack in the envelope.

3. Securely close the aluminum envelope, place it in the white box.
4. Fill the **SPECIMEN TRANSPORT FORM** and put it in the box. Tape the outside of the white shipping box.
5. **Drop the Package containing the specimens at the clinic** in the next 12-24 hours. No later than 48 hours.

C. STOOL PACKAGING INSTRUCTIONS: Shipping via FedEx to CBRD Central Lab

1. Place the gel packs in the bottom of the aluminum envelope provided.
2. Place the sealed collection bag on top of the gel pack in the envelope.
3. Securely close the aluminum envelope, place it in the white box with blue "List of Contents" card.
4. Fill the **SPECIMEN TRANSPORT FORM** and put it in the box. Tape the outside of the white shipping box.
5. Adhere the **Fedex shipping label** to the outside of the box.
6. **Drop the Package at a Fedex location** in next 12-24 hours. No later than 48 hours. It could be mailed on ***MONDAY, TUESDAY, WEDNESDAY.***
7. Send shipment notification to eppic-netbiorepository@nyulangone.org and log shipment in the EPPIC-Net Portal - Biorepository page, LabVantage link.

1. Fully complete the barcoded labels on cryovials marked "Surgery", please select the correct one depending on the spinal tissue type*.
2. Weigh the empty cryotube (record the tube mass before adding tissue) at the lab.
3. Right after surgical removal, place the surgical spinal tissue* into a sterile cup with 10 ml of PBS. This step is to remove blood product from the tissue.

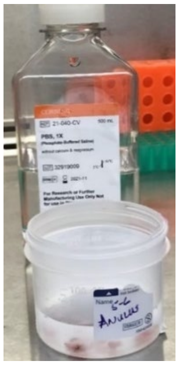

4. Remove the spinal tissue from the cup and blot it on sterile gauze to remove excess liquid.

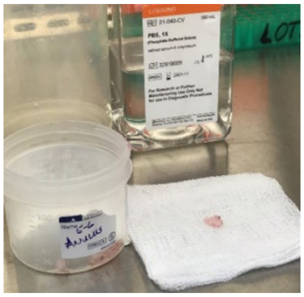

5. And immediately place the tissue into the labeled 5mL cryotube. Immerse the cryotube in a bucket containing white ice. Rush to the laboratory, where the specimen is going to be stored.
   Note: steps 3-5 should take no longer than 15 minutes.

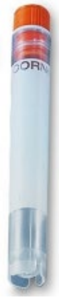

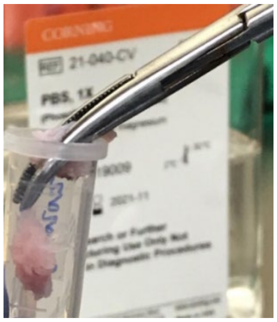

6. Dry the outside of the cryovial with a kim-wipe. Weigh the tube to get tissue mass = (weight of tube + tissue) – (weight of empty tube).
7. Immerse the cryotube in a liquid nitrogen container to flash freeze.
8. Store in a -80° C freezer until shipment to CBRD Central Laboratory.
9. Ship frozen on Dry ice according to Batch shipment schedule using Dry ice shipper with Shipping manifest.

10. Email shipment notification to eppic-netbiorepository@nyulangone.org and log shipment in the EPPIC-Net Portal - Biorepository page, LabVantage WebAccess.

* Spinal tissue types

a. Paraspinal muscle
b. Ligmentum flavum
c. Vertebral bone
d. Facet cartilage
e. Disc endplate
f. Disc annulus fibrosus
g. Disc nucleus pulposus (if young, then NP will swell up with PBS wash and increase in mass).

Sample Requisition Forms: BACPAC

PATIENT-SPECIFIC COLLECTON SHEETS

- Below are the samples of what the requisition forms will look like for biofluids collection/tissue collection.
- Please print out a copy every time samples will be shipped to NYU CBRD. Make sure the collection sheet is filled out completely with the correct information.
- Ensure correct patient requisition label is placed on the requisition form.
- Send back any unused labels to CBRD as well.
- Label sample aliquots in numerical order following the S-ID #'s on the bottom of the tube/aliquot labels. Send the aliquots in numerical order to CBRD as well.

NYU Langone Health

NYU LANGONE CENTER FOR BIOSPECIMEN RESEARCH & DEVELOPMENT:
EPPIC-Net: BACPAC

BIOSPECIMEN REQUISITION FORM

PROTOCOLID: ______

Patient Number				
Date of Collection Example: 01 JAN 2001				
Site				
Visit (check one ONLY one visit)	☐ Baseline	☐ 12 mo	☐ 24 mo	☐ 36 mo
Requisition Completed by				

SPECIMEN	# OF TUBES DRAWN (mark one)	COLLECTION TIME (HH:MM)	ALIQUOTS YIELDED/Site Distribution (fill out)	TIME STORED (-80°C)
EDTA PLASMA*	☐ 1x10mL<br>☐ N/A		_____Site aliquots (0.5mL)<br>_____CBRD aliquots (0.5mL)	
SST SERUM*	☐ 1x10mL<br>☐ N/A		_____Site aliquots (0.5mL)<br>_____CBRD aliquots (0.5mL)	
PAXgene RNA WHOLE BLOOD	☐ 2x2.5mL<br>☐ Other:___x2.5mL		____Site RNA PGX (2.5mL)<br>____CBRD RNA PGX (2.5mL)	
SALIVA	☐ 2x2mL<br>☐ Other:___x2mL		____Site Saliva Tube (2 mL)<br>____CBRD Saliva Tube (2mL)	
URINE*	☐ 1x10mL<br>☐ N/A		_____Site aliquots (4 mL)<br>_____CBRD aliquots (4 mL)	
STOOL	☐ 2x4mL<br>☐ Other:___x4mL		____Site Stool Tube (4 mL)<br>____CBRD Stool Tube (4mL)	

EXAMPLE

COMPLETED *(dd-mm-yyyy):* ______________________

* = has aliquots

PACKED *(dd-mm-yyyy):* ______________________

SHIPPED *(dd-mm-yyyy):* ______________________

FED-EX WAYBILL #: ______________________

NYU LANGONE CENTER FOR BIOSPECIMEN RESEARCH & DEVELOPMENT:
EPPIC-Net: BACPAC

BIOSPECIMEN REQUISITION FORM **PROTOCOLID: ______**

**Patient Number**	
**Date of Collection** Example: 01 JAN 2001	
**Site**	
**Visit**	**Surgery**
**Requisition Completed by**	

SPECIMEN	COLLECTION TIME (HH:MM)	TISSUE MASS	ALIQUOTS YIELDED/Site Distribution (fill out)	TIME STORED (-80°C)
PARASPINAL MUSCLE		______ WEIGHT OF EMPTY TUBE<br>______ WEIGHT OF TUBE + TISSUE<br>______ WEIGHT OF TISSUE	____Site aliquots<br>____CBRD aliquots	
LIGMENTUM FLAVUM		______ WEIGHT OF EMPTY TUBE<br>______ WEIGHT OF TUBE + TISSUE<br>______ WEIGHT OF TISSUE	____Site aliquots<br>____CBRD aliquots	
VERTEBRAL BONE		______ WEIGHT OF EMPTY TUBE<br>______ WEIGHT OF TUBE + TISSUE<br>______ WEIGHT OF TISSUE	____Site aliquots<br>____CBRD aliquots	
FACET CARTILAGE		______ WEIGHT OF EMPTY TUBE<br>______ WEIGHT OF TUBE + TISSUE<br>______ WEIGHT OF TISSUE	____Site aliquots<br>____CBRD aliquots	
DISC ENDPLATE		______ WEIGHT OF EMPTY TUBE<br>______ WEIGHT OF TUBE + TISSUE<br>______ WEIGHT OF TISSUE	____Site aliquots<br>____CBRD aliquots	
DISC ANNULUS FIBROSUS		______ WEIGHT OF EMPTY TUBE<br>______ WEIGHT OF TUBE + TISSUE<br>______ WEIGHT OF TISSUE	____Site aliquots<br>____CBRD aliquots	
DISC NUCLEUS PULPOSUS		______ WEIGHT OF EMPTY TUBE<br>______ WEIGHT OF TUBE + TISSUE<br>______ WEIGHT OF TISSUE	____Site aliquots<br>____CBRD aliquots	

EXAMPLE

COMPLETED *(dd-mm-yyyy):* ____________________

PACKED *(dd-mm-yyyy):* ____________________

SHIPPED *(dd-mm-yyyy):* ____________________

FED-EX WAYBILL #: ____________________

BARCODE
LABEL HERE

NYU LANGONE CENTER FOR BIOSPECIMEN RESEARCH & DEVELOPMENT
EPPIC-Net: BACPAC

BIOSPECIMEN REQUISITION FORM – STOOL

To be completed by Site:

Subject Number	
Visit	☐ Baseline
Requisition Completed by:	

To be completed by Participant:

Date of Birth<br>(Example: 01/01/2021)	
Date of Collection<br>(Example: 01/01/2021)	
Time of Collection<br>(Example: 01:01)	
Date of Shipment<br>(Example: 01/01/2021)	
Fed-Ex Air WayBill Tracking #<br>(XXXX XXXX XXXX)	

Specimen	# Tubes Collected
DNA/RNA Shield Fecal Collection	☐ 1 x 9 mL

To be completed by CBRD:

Date Received	# Tubes Received	Date Stored at -80° C

NYULMC CBRD | 550 First Avenue |Medical Science Building |Laboratory 239 |New York, NY 10016

SHIPPING SPECIMENS TO CBRD CENTRAL LABORATORY

PACKAGING PROCEDURES: FROZEN BIOLOGICAL SUBSTANCE, CATEGORY B

DO NOT ship specimens on Fridays, Holidays, or WITHOUT COMMUNICATION and CONFIRMATION from the CBRD Central Laboratory

- Batched Samples are to be shipped once quarterly
- Complete the Shipping Manifest Form electronically or by hand and attach it in EPPIC-Net DCC LabVantage WebAccess shipment notification

  Follow IATA PI 650/UN3373 Regulations for Biological Substance Catogory B Shipments

1. Packaging Procedure
   a. Insert the tubes into a resealable biospecimen transport bag or tamper evident 95kPa biospecimen bag containing an absorbent pack. Seal the bag, remove air by pressing from center to edge to seal.
   b. Fold the completed requisition form and place it into the pocket on the reverse side of the specimen collection bag. **The bar code must be visible.** Include a copy of the shipping manifest.

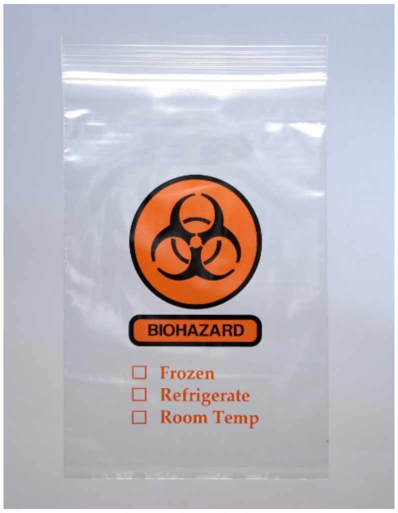

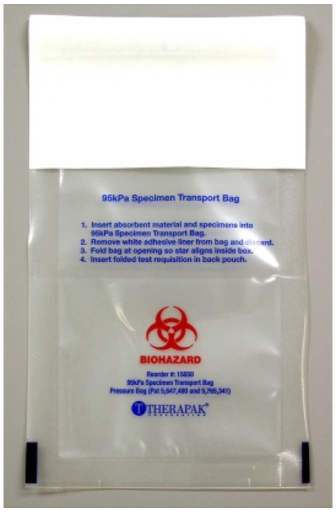

   c. Fill half of the Styrofoam box with dry ice and insert the specimen collection bag(s).

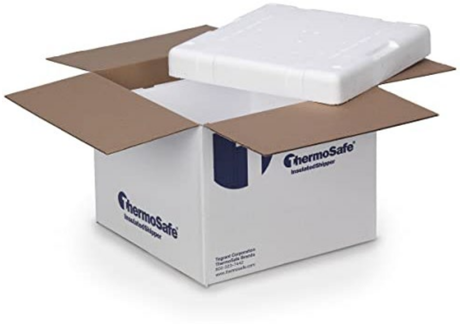

d. Fill the container up with dry ice, replace the Styrofoam lid.
e. Seal the outside cardboard box and ensure a tight seal.
f. Place FedEx airway bill on top of box.
g. Affix **UN3373 Human Biological Substance label** and **Dry Ice label** to exterior of *box-BE SURE TO COMPLETE weight and shipper/consignee section on dry ice label*

It is ***essential*** that the samples **never** be thawed once frozen at -80° C. Samples therefore ***must*** be shipped on a sufficient amount of dry ice.

- **SHIP SPECIMENS TO CBRD CENTRAL LABORATORY**

Center for Biospecimen Research and Development (**CBRD**) NYU Langone Medical Center

550 1st Avenue, Medical Science Building, room 238
New York, New York 10016
eppic-netbiorepository@nyulangone.org
PH: 1 (646) 501-4268
Fax: 1 (646) 501-4579
